# ALIGNING GERIATRIC EMERGENCY CARE WITH THE 4MS OF THE AGE-FRIENDLY HEALTH SYSTEM FRAMEWORK

**DOI:** 10.1093/geroni/igac059.1336

**Published:** 2022-12-20

**Authors:** Jennifer Raymond

**Affiliations:** Dartmouth-Hitchcock Medical Center, Lebanon, New Hampshire, United States

## Abstract

In 2019 Dartmouth-Hitchcock Medical Center (DHMC) was recognized by the Institute for Healthcare Improvement as a level 2 Age Friendly Health System (AFHS). Over the past two years DHMC leveraged the strengths of the AFHS to become a level 1 American College of Emergency Physicians (ACEP) accredited geriatric emergency department. Leveraging the paradigm of 4M care including: What Matter’s Most, Mobility, Medication and Mentation, DHMC achieved alignment between the geriatric emergency department accreditation and the AFHS framework. Pathways focusing on the 4Ms have been identified as a model to improve transitions of care post ED. This talk will focus on lessons learned and outcome data specific to the creation of the 4M pathways and transitions of care,

